# An Open-Label Prospective Study to Compare the Efficacy and Safety of Topical Fluticasone Versus Tacrolimus in the Proactive Treatment of Atopic Dermatitis

**DOI:** 10.5826/dpc.1004a94

**Published:** 2020-10-26

**Authors:** Vinodhini R. Mudaliyar, Asha Pathak, Alok Dixit, Sweta S. Kumar

**Affiliations:** 1 Department of Pharmacology, Uttar Pradesh University of Medical Sciences, Saifai, Etawah, India; 2 Department of Dermatology, Uttar Pradesh University of Medical Sciences, Saifai, Etawah, India

**Keywords:** topical corticosteroids, topical calcineurin inhibitors, fluticasone propionate, tacrolimus, atopic dermatitis

## Abstract

**Background:**

Atopic dermatitis (AD), a chronic, recurrent inflammatory skin condition primarily affects children. Topical treatment, systemic treatment, and phototherapy are mainstays of treatment. Topical corticosteroids (TCS) are first-line therapy for AD but are associated with various adverse effects. Topical calcineurin inhibitors (TCI) can be used as an alternative to TCS.

**Objective:**

The aim of the study is to compare the efficacy of topical preparations of fluticasone and tacrolimus in reducing the severity of disease and, to assess the quality of life (QoL), and to estimate if any association exists between severity of disease and QoL.

**Methods:**

Thirty-seven children with AD randomly received one of the 2 topical treatments, with daily application for the first 4 weeks in the acute phase and twice weekly for next 4 weeks in the maintenance phase. The severity of disease was assessed using SCORing Atopic Dermatitis (SCORAD), and QoL was assessed using the Children’s Dermatology Life Quality Index (CDLQI).

**Results:**

At the end of the acute phase, there was a reduction in SCORAD score of 69.29% in the fluticasone group and 64.2% in the tacrolimus group (P < 0.001). In the maintenance phase, the score had risen in the fluticasone group by a mean difference of 0.81, while in the tacrolimus group it decreased by 0.99. Both fluticasone and tacrolimus groups improved in children’s QoL (P < 0.001). Positive correlation (r = 0.4668) exists between SCORAD and QoL. The most common adverse skin reaction noted was skin burning with tacrolimus.

**Conclusions:**

Fluticasone and tacrolimus are equally efficacious in the treatment of AD and have similar benefits with children’s QoL. Tacrolimus is better than fluticasone at reducing the extent of lesions.

## Introduction

Atopic dermatitis (AD) is a chronic, recurrent inflammatory skin condition, characterized by acute flare-ups of pruritic lesions, afflicting 15%–20% of children in developed countries [1]. It has no known cure and adversely affects the quality of life (QoL) of patients and their families [2,3]. Management of AD includes identifying and eliminating the triggering factors, restoring the skin barrier function, reducing inflammation, and treating secondary infection with antibiotics. Among topical treatments there are bathing, emollients, topical corticosteroids (TCS), and topical calcineurin inhibitors (TCI) [4]. Proper bathing followed by the regular use of skin emollients for restoring hydration and identifying and eliminating trigger factors may be helpful in the treatment of acute flare-ups, but they do not sufficiently suppress the inflammation occurring deep at the molecular level to prevent future exacerbations. Topical treatments like TCS [5,6] and TCI [7,8] help to reduce inflammation by blocking various inflammatory cytokine productions at different levels of pathophysiology. Both TCS and TCI are first-line therapy for AD and are proven to be useful in both acute flare-ups and maintenance therapy. However, the major drawback encountered with TCS is the adverse effects associated with chronic use. TCI are an excellent alternative with no steroid-related side effects, and they are safer to use on thinner skin [9]. They are either used alone or in combination with TCS and have proven to reduce frequency and severity of flare-ups.

Although TCS and TCI are routinely prescribed topical treatments for AD in outpatient settings, TCI are often selected for patients who do not respond to TCS and when the proactive approach is not a routinely followed strategy. Available clinical studies neither reflect the common clinical practice nor the need for long-term treatment of AD in the Indian population. A study by Berth-Jones et al [10] compared different formulations of fluticasone with placebo in a 16-week proactive regimen. After the amelioration in the acute phase, the risk of AD flare was 1.9 times lower with fluticasone ointment than under placebo. Breneman et al [11] in a randomized study tested the proactive use of tacrolimus 0.03% ointment 3 times weekly in children that showed a longer median flare-free period in the proactive treatment group than in the control group. The differences in the study population, drug application schedule, and study duration make a direct comparison with various drugs a difficult task.

We chose to study TCS and TCI as first-line treatment both for reactive and proactive therapy. We divided our study into 2 phases in order to assess the efficacy and safety of topical treatment in the acute flare-up phase followed by a 4-week maintenance phase. The primary objective of the study was to compare the efficacy of topical preparations of 0.005% fluticasone versus 0.03% tacrolimus ointment in the treatment of AD. The secondary objective was to assess the QoL, to find the association between severity of disease and QoL, and to assess the safety and tolerability of the medications.

## Methods

### Study design

The present study was an 8-week, prospective, randomized, and an open-label study conducted on 37 children with AD in the Department of Pharmacology in collaboration with the Department of Dermatology, UPUMS Saifai, Etawah. The study was approved by the Institutional Ethics Committee (EC no. 39/2018), and written informed consent from the childrens’ parents was obtained after explaining the nature of the proposed medical intervention and reasonable consequences of their participation in the study.

Thirty-seven children who met the inclusion criteria (age 2–16 years of either gender, meeting the Hanifin-Rajka clinical criteria for the diagnosis of AD [12]) were recruited for the study period of 56 days (a 4-week acute flare-up phase followed by a 4-week maintenance phase). Any form of dermatitis other than AD, untreated bacterial, fungal or viral skin lesion, or history of allergic reaction in the past to topical medications employed in this study were excluded. All 37 children were randomly assigned in 1:1 ratio to each group. Group 1 (n = 19) received 0.005% fluticasone ointment twice daily and group 2 (n = 18) received 0.03% tacrolimus ointment once daily at night before sleep. In the maintenance phase (n=35), both groups continued the same topical medication twice weekly on 2 consequent nights at bedtime. Medicines were applied using the fingertip unit (FTU) method [13].

The primary outcome comparing the efficacy of the 2 drugs was measured by the SCoring Atopic Dermatitis (SCORAD) index [14]. The SCORAD index consisted of the description of the extent, intensity, and subjective symptoms. The formula applied for the SCORAD index was: A/5 + 7B/2 + C. We used a body diagram to analyze the area affected, and the most representative lesion was used for scoring. The intensity element of the SCORAD index consisted of 6 items: erythema, edema/papules, the effect of excoriations, lichenification, oozing/crust formation, and dryness, and each item had 4 grades: 0, 1, 2, and 3. Dryness was assessed in non-inflamed skin. During the last 3 days, subjective items, that included daily pruritus and sleeplessness, were graded on a 10-cm visual analogue scale drawn on the SCORAD form.

The secondary outcome determining the change in the QoL after treatment was analyzed by the Children’s dermatology life quality index (CDLQI). [15] Both SCORAD and CDLQI scores were noted on the day of enrollment and at the end of weeks 2, 4, 6, and 8.

### Statistics

Age and gender values of recruited children were analyzed with the chi-square test. Data analysis for efficacy, as measured by SCORAD and QoL of AD children using the CDLQI index, was performed by repeated measures ANOVA. The association between SCORAD and CDLQI was conducted by Pearson’s correlation and linear regression. The analysis was conducted on SPSS software, and a P value of <0.05 was considered statistically significant.

## Results

Table 1 shows the baseline demographic data pf gender details, age-wise distribution of cases, extent, intensity, subjective symptoms, SCORAD and QoL scores. We screened a total of 37 children with moderately active AD. The mean ±SD age of all 37 children recruited for the study was 10.13 ± 3.61 years. Group wise (mean ± SD) age was 9.84 ± 3.78 years and 10.44 ± 3.50 years with no significant difference between groups. Approximately 81% of children with AD had a personal or family history of atopy. At the end of the acute phase, 35 children entered into the 4-week maintenance phase; 2 children from group 1 discontinued the study, as they were satisfied with lesion improvements and did not show interest in continuing the treatment for one additional month.

**Figure 1:**
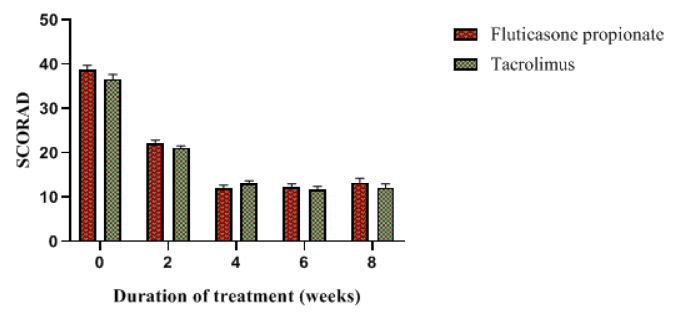
Changes in SCORAD in both treatment groups at week 0 (baseline), 2, 4 (end of acute phase), 6, and 8 (end of maintenance phase) [Mean ± SEM]. SCORAD = scoring atopic dermatitis.

**Figure 2:**
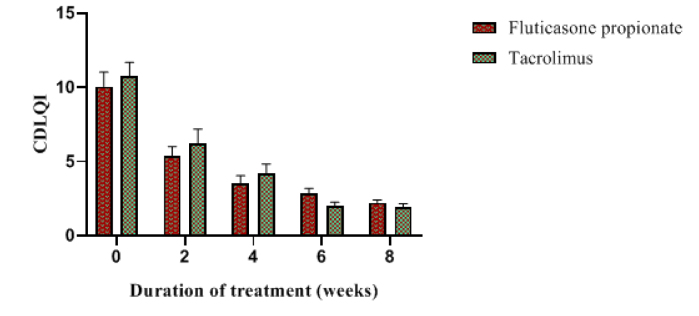
Changes in CDLQI in both treatment groups at week 0 (baseline), 2, 4 (end of acute phase), 6, and 8 (end of maintenance phase) [Mean ± SEM]. CDLQI = children’s dermatology life quality index.

Figures 1 and 2 show the changes in SCORAD and CDLQI in both groups. The intergroup comparison of SCORAD and CDLQI at respective baseline and all follow-up intervals were comparable. In the acute phase treatment, there was a reduction in the SCORAD by 42.60% and 69.29% at weeks 2 and 4 visits respectively when compared to baseline in the fluticasone group and by 42.34% and 64.20% in the tacrolimus group. Significant reductions (P < 0.001) were observed in both SCORAD and CDLQI at week 2 and at end of the acute phase in both treatment groups. In the maintenance phase, no significant change in SCORAD was observed in either of the treatment groups. However, the change in CDLQI was significant (P < 0.05) at week 8 when compared to baseline (week 4) within the fluticasone group, and in the tacrolimus group the change was significant at weeks 6 and 8 when compared to baseline (P < 0.05).

**Figure 3:**
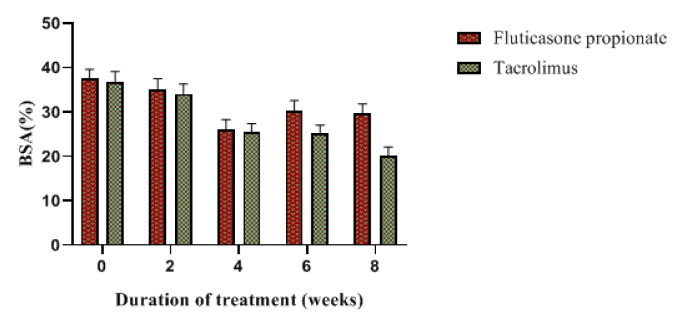
Changes in the extent of lesions (BSA) in both treatment groups at week 0 (baseline), 2, 4 (end of acute phase), 6, and 8 (end of maintenance phase) [Mean ± SEM]. BSA = body surface area.

**Figure 4:**
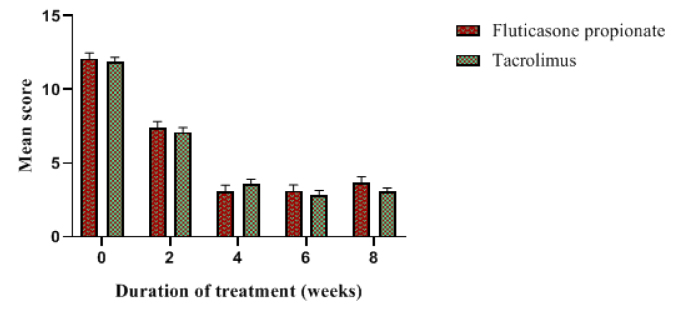
Changes in the subjective symptoms in both treatment groups at week 0 (baseline), 2, 4 (end of acute phase), 6, and 8 (end of maintenance phase) [Mean ± SEM].

Figures 3 and 4 show changes in the body surface area (BSA) and subjective scores. The mean extent of lesions and subjective symptoms at the time of recruitment was comparable between both treatment groups. However, there was significant (P < 0.05) reduction in the tacrolimus group at week 8 than in the fluticasone group. At the end of the acute phase, there was significant reduction of mean extent and subjective scores at all follow-up visits in both treatment groups. In the maintenance phase, a significant mean difference for subjective symptoms was observed within the tacrolimus group at week 6.

**Figure 5:**
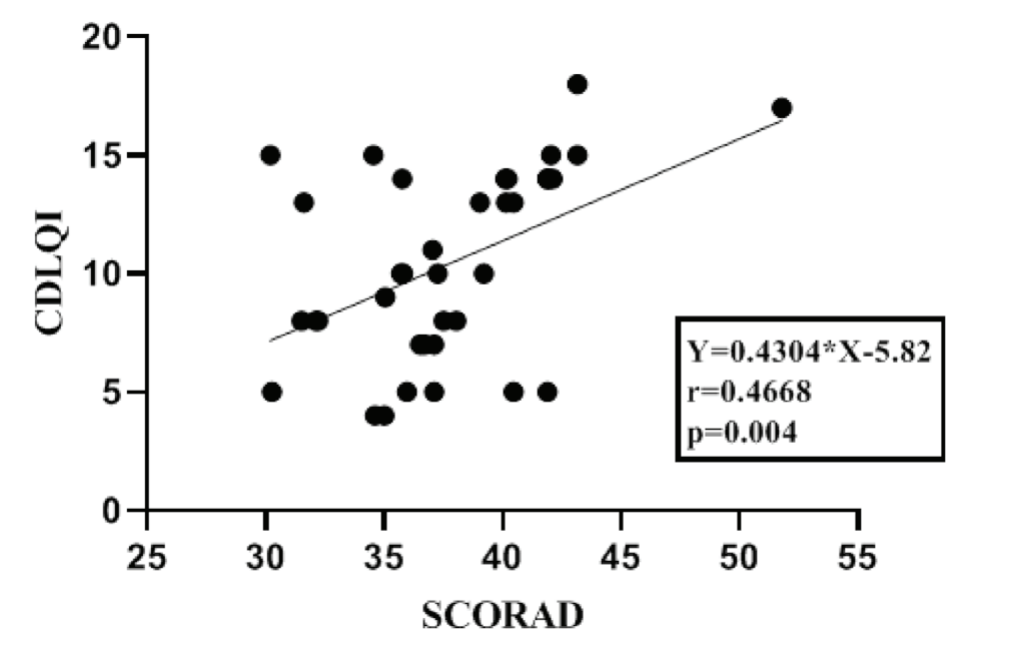
Association between SCoring atopic dermatitis (SCORAD) and children’s dermatology life quality index (CDLQI).

Figure 5 shows the association between the severity of the disease (SCORAD) and CDLQI. The analysis revealed statistical significance with P = 0.004, indicating the existence of a positive moderate correlation (r = 0.4668) between the 2 instruments. The most common adverse drug reaction (ADR) was a sensation of skin burning. It was observed in 3 (8%) of all children receiving tacrolimus. No ADR was found with the use of fluticasone.

## Discussion

This study evaluates the efficacy of an intermediate potent TCS, fluticasone 0.005% ointment and a TCI, tacrolimus 0.03% ointment for treatment of moderate to severe AD in Indian children. In this study, we included a total of 37 children with moderately active AD. The boy-to-girl ratio in the present study was 1.31:1, which is comparable to a clinico-epidemiological study of AD in North India, where male predominance was noted with 2.25:1 in infants and 1.3:1 in children [16].

The age distribution within 0–5 years, 6–10 years, and ≥11 years were 4, 7, 8 and 2, 7, 9 children in the 2 groups respectively. There was a greater proportion of children within the ≥ 11-years age group, although the disease is known to begin early in age. About 65% develop symptoms at 1 year of age, and 90% of affected individuals are younger than 5 years [17]. The disparity in the age of presentation in our study could be due to the inclusion criteria of recruiting children younger than 2 years of age.

The present study found that both treatment groups had a significantly reduced SCORAD as early as week 2. At week 2 follow-up, only 1 child had moderate AD in the fluticasone group. In the rest of the children, SCORAD had decreased into the mild severity range. The results of this study show that both fluticasone and tacrolimus had similar efficacy in the acute treatment phase. In the maintenance phase, the SCORAD had risen in the fluticasone group by a mean difference of 0.8, while in tacrolimus it decreased by 0.99. Due to the lack of head-to-head comparison of TCS and TCI, it was difficult for us to compare our results with previous reports. However, our result is comparable to the longer median time to flare-up seen in 2 studies using fluticasone and tacrolimus separately [10,11] at approximately 16 weeks with use of fluticasone and 24 weeks with tacrolimus ointment. However, the disease severity in our study population was maintained in low range in both treatment groups, and there were no flare-up events observed within 8 weeks. Clinically and statistically speaking, the difference in SCORAD was not significant in the present study, and it can be concluded that both treatments are efficacious during the intermittent maintenance phase to prevent acute exacerbations.

A randomized, multicenter study determining the anti-pruritic efficacy of 0.03% topical tacrolimus (for children <16 years old) and 0.1% (for adults >16 years old) reported a mean SCORAD decrease from 29.1 to 17.3 with a mean difference of 11.8 (P < 0.0001) after 4 weeks [18]. In another study where 0.1% tacrolimus ointment was given twice a day, the drug showed 20.72% and 64.05% reduction in SCORAD at the end of weeks 2 and 4 respectively [19]. The efficacy in our study was approximately the same at week 4. However, at week 2, we had a greater SCORAD reduction by a difference of 21.62%. Similarly, the SCORAD reduction was greater by a difference of 23.65% at week 4 compared to the earlier study. The disparity in the results could be due to the different concentrations of ointment used and the selection of the study population.

In a study conducted by Doss et al [20] on 479 children with AD who had responded insufficiently to conventional TCS treatment, fluticasone 0.005% ointment and tacrolimus 0.03% ointment were used as second-line treatment. The response rate was 86.3% in the tacrolimus group and 91.5% in the fluticasone group at day 21. This result was comparable to our study, as we had an approximately equal reduction in severity between both treatment groups.

AD is known to affect the QoL of patients and their family members. A majority of studies use SCORAD, but there are limited studies that try to find the association between severity of disease and QoL. The present study tried to assess the QoL of children and analyze the association between the severity of the disease and children’s QoL.

The association between the SCORAD and CDLQI indices showed reasonable positive moderate correlation (r = 0.4668) with P = 0.004. A similar study done by Kim et al [21] showed positive correlation between the SCORAD index and CDLQI (r = 0.312, P = 0.039). Another study by Bezerra Campos et al [22] found significant positive correlation (r = 0.68, P < 0.001) between severity of disease and QoL of pediatric patients with AD. Our study had similar comparable results and indicates the importance of including the assessment of QoL as an objective to the clinical analysis. The result of the present study demonstrates that the QoL is related to the severity of AD. The higher the SCORAD, the poorer the QoL; therefore, early management of AD may help improve the QoL of AD patients. In addition to medical management, psychological support may improve the long-term physical and emotional outcomes of all AD sufferers. The QoL of children improved dramatically in both the treatment groups and continued to improve even through the maintenance phase.

In our study, of all children with AD, 3 (8%) of those receiving tacrolimus treatment developed ADR. The ADR observed was a mild and transient application site reaction. In a 12-week study of both 0.03% and 0.1% tacrolimus by Paller et al [23], the most common ADRs observed were application site reactions, particularly skin burning and pruritus. They appeared during the first few days and then declined. Most episodes of a skin-burning sensation lasted less than 15 minutes. Similarly, most episodes of pruritus lasted less than 2 hours.

## Limitations

Our study has certain limitations, and the findings in this study should be interpreted with caution. First, the study had no wash-out period, and the drugs were initiated on the first visit to the outpatient department once the diagnosis was made. Second, since the scoring system used for outcome measures included objective and subjective symptoms, the final score may have been influenced by parents, guardians, or by the children themselves. Furthermore, we did not estimate cost effectiveness. The other shortcomings of the study are the small sample size and short study duration in the maintenance phase. Thus, it is not possible to convincingly comment on time to flare-up and the long-term safety of both treatments. Another demerit is that it was an open-label study. A longer duration, single or double-blind study, and a larger sample size would be helpful in covering these shortcomings.

## Conclusions

Both fluticasone and tacrolimus are equally efficacious in reducing severity of disease and preventing flare-ups in children suffering from moderate to severe AD. At week 8, tacrolimus is more efficacious than fluticasone in improving the extent of AD. However, in both groups the fluticasone and tacrolimus were equally efficacious in reducing subjective scores and improving QoL in AD patients. Positive correlation exists between the severity of the disease and children’s quality of life.

## Figures and Tables

**Table 1:** Demographic and Disease Characteristics in Children With Atopic Dermatitis

Treatment Groups	Fluticasone Group (n=19)	Tacrolimus Group (n=18)
Male:Female	12:7	9:9
2–5 years (M/F)	2/2	1/1
6–10 years (M/F)	4/3	2/5
≥11 years (M/F)	6/2	6/3
Age years ± SD	9.84 ± 3.78	10.44 ± 3.50
**Affected sites—number of children that presented the body area involved in AD**		
Head and neck	8	3
Extremities	15	14
Trunk and back	13	15
Genitals	3	3
**Total affected BSA**		
BSA ± SD	37.57 ± 8.94	36.66 ± 10.35
0 to ≤25%	2	0
>25 to ≤50%	13	3
>50 to ≤75%	4	9
>75 to ≤100%	0	6
**Severity of AD**		
SCORAD	38.75 ± 4.13	36.60 ± 4.45
Mild (<25)	1	2
Moderate (25 to ≤50)	16	14
Severe (>51)	2	2
**Intensity**		
Erythema	1.78 ± 0.71	1.55 ± 0.70
Edema/papules	0.89 ± 0.73	0.66 ± 0.59
Oozing/crusting	0.78 ± 0.63	0.88 ± 0.67
Excoriation	0.63 ± 0.76	0.55 ± 0.61
Lichenification	0.52 ± 0.51	0.50 ± 0.70
Dryness	1.84 ± 0.68	1.83 ± 0.51
**Subjective symptoms**		
Pruritus	6.10 ± 0.99	5.83 ± 0.70
Sleeplessness	6.00 ± 0.94	6.05 ± 0.63
**Quality of life**		
CDLQI	10.05 ± 4.27	10.77± 3.84
None or small	11	9
Moderate	8	9
Severe	0	0
**Domains of the CDLQI**		
Symptoms/feelings	3.10 ± 1.24	3.05 ± 1.25
Leisure	2.05 ± 1.80	2.44 ± 1.58
School/holidays	0.84 ± 0.68	0.83 ± 0.78
Relationships	2.00 ± 1.41	2.22 ± 1.00
Sleep	1.73 ± 0.80	1.44 ± 0.61
Treatment	0.31 ± 0.47	0.77 ± 0.64
**Medical history—number (%) of children with known trigger factors**		
Personal and family history of atopy	15 (78.94)	15 (83.33)
Seasonal variations (%)	11 (57.89)	12 (66.66)

AD = atopic dermatitis; BSA = body surface area; SCORAD = scoring atopic dermatitis; CDLQI = children’s dermatology life quality index.
